# Grassland harvesting alters ant community trophic structure: An isotopic study in tallgrass prairies

**DOI:** 10.1002/ece3.5523

**Published:** 2019-08-13

**Authors:** Tania Kim, Savannah Bartel, Claudio Gratton

**Affiliations:** ^1^ Great Lakes Bioenergy Research Center University of Wisconsin Madison Madison WI USA; ^2^ Department of Integrative Biology University of Wisconsin Madison Madison WI USA; ^3^ Department of Entomology University of Wisconsin Madison Madison WI USA

**Keywords:** disturbance, niche space, stable isotopes, trophic position, trophic range

## Abstract

Disturbances have long been recognized as important forces for structuring natural communities but their effects on trophic structure are not well understood, particularly in terrestrial systems. This is in part because quantifying trophic linkages is a challenge, especially for small organisms with cryptic feeding behaviors such as insects, and often relies on conducting labor‐intensive feeding trials or extensive observations in the field. In this study, we used stable isotopes of carbon and nitrogen to examine how disturbance (annual biomass harvesting) in tallgrass prairies affected the trophic position, trophic range, and niche space of ants, a widespread grassland consumer. We hypothesized that biomass harvest would remove important food and nesting resources of insects thus affecting ant feeding relationships and trophic structure. We found shifts in the feeding relationships inferred by isotopic signatures with harvest. In particular, these shifts suggest that ants within harvest sites utilized resources at lower trophic levels (possibly plant‐based resources or herbivores), expanded trophic breadth, and occupied different niche spaces. Shifts in resource use following harvest could be due to harvest‐mediated changes in both the plant and arthropod communities that might affect the strength of competition or alter plant nitrogen availability. Because shifts in resource use alter the flow of nutrients across the food web, disturbance effects on ants could have ecosystem‐level consequences through nutrient cycling.

## 1 INTRODUCTION

Disturbances have long been recognized as important forces for structuring natural communities (Connell, 1978; Dayton, 1971; Sousa, 1984). Disturbances can increase or decrease species diversity depending on their severity, timing, and spatiotemporal extent and can also affect ecological functions such as nutrient cycling, primary productivity, seed dispersal, and pollination (Markl et al., 2012; Thom & Seidl, 2016; Tilman et al., 2000). However, the impact of disturbance on trophic structure (the organization of species within a food web) is not as well understood, particularly in terrestrial systems. This is, in part, because determining feeding relationships and tracking the flow of nutrients within food webs is logistically challenging, especially with organisms with cryptic feeding behaviors. Because characterizing the trophic structure of a community can shed light on the ecological function and niche use of different species (beyond community‐wide metrics such as species richness and abundance), understanding the impact of disturbance on trophic structure can provide insight into community assembly processes and resilience to subsequent disturbance events (Biswas & Mallik, 2010; Cardinale & Palmer, 2002; McCann, 2000; Thom & Seidl, 2016).

Disturbances are expected to affect trophic structure and trophic interactions by affecting the abundance and occurrence of species at multiple trophic levels. For example, if disturbances affect resource abundance and composition, then consumers may alter their feeding through frequency‐dependent prey switching or may truncate or expand their diet breadth based on the availability of their preferred prey (Jaworski, Bompard, Genies, Amiens‐Desneux, & Desneux, 2013; Murdoch, 1969; Resasco, Levey, & Damschen, 2012). In contrast, if disturbances alter consumer abundance and composition, these changes could affect trophic structure through competition (Wootton, 1998). For example, if a disturbance reduces the abundance of a dominant competitor, then this may alleviate competition between consumers and allow subordinate species to broaden their diet breadth (Fründ, Dormann, Holzschuh, & Tscharntke, 2013; Inouye, 1978; Pacala & Roughgarden, 1982; Spiesman & Gratton, 2016). Because changes in the feeding behavior of consumers (whether mediated through resources or consumer competition) ultimately alter the flow of nutrients through food webs, disturbance effects on trophic interactions and structure can scale up to affect ecosystem‐level processes, such as nutrient cycling, as well.

In human‐managed habitats such as grasslands, management actions such as haying, fire, and grazing, create disturbances by removing aboveground biomass that can otherwise serve as important food and shelter resources for animals. Management actions are likely to affect the feeding behavior of insects, but documenting feeding behavior is a challenge and often relies on conducting extensive feeding trials and observations in the field. For small and cryptic organisms, such as insects, this presents a logistical challenge and thus indirect measures are needed. Stable isotope ratios can be used to infer trophic structure as they provide time‐integrated measures of energy flow within food web and are commonly used in aquatic and terrestrial systems (Vander Zanden, Casselman, & Rasmussen, 1999; Vander Zanden, Olden, Gratton, & Tunney, 2016). Specifically, the isotopic ratios of nitrogen (^15^N/^14^N) are often used to determine the trophic position of consumers because δ^15^N is enriched with trophic transfers up a food chain (Fry, 2006). In contrast, the isotopic ratios of carbon (^13^C/^12^C) are largely conserved within the food chains, and therefore, δ^13^C is used to identify the source of a consumer's resource base. Comparing changes in δ^13^C and δ^15^N in the presence and absence of disturbances can reveal how trophic structure (e.g., trophic breadth, trophic position) might change following a disturbance.

In this study, we examined how annual harvesting of tallgrass prairies in southern Wisconsin (USA) affected the trophic structure of grassland ants as inferred by analyses of naturally occurring stable isotope patterns. Specifically, we asked how annual harvesting of grasslands affects (a) δ^15^N and δ^13^C of baseline plant resources, and (b) community‐wide measures of trophic structure derived from stable isotopes (trophic position, trophic range, isotopic niche space). To address possible mechanisms underlying harvest effects, we asked (c) whether site‐level differences in soil isotopic signatures, insect herbivore abundances, and ant abundances correlate with changes in ant trophic structure. We focus on ants as consumer species because they have diverse diets including plant‐derived material such as seeds, nectar, and honeydew from sucking insects, and animal‐derived materials including herbivores, predators, and microarthropods such as collembola and springtails. Ant species have been shown to vary in isotopic signatures of N and C (Blüthgen, Gebauer, & Fiedler, 2003; Fiedler, Kuhlmann, Schlick‐Steiner, Steiner, & Gebauer, 2007; Ponsard & Arditi, 2000; Tillberg, McCarthy, Dolezal, & Suarez, 2006) reflecting their varying ecological roles in different natural and managed systems (Gibb & Cunningham, 2011; Mooney & Tillberg, 2005; Ottonetti, Tucci, Chelazzi, & Santini, 2008). While there are a few studies that have tested whether disturbance affects trophic structure of ants (e.g., Penick, Savage, & Dunn, 2015; Resasco et al., 2012; Woodcock et al., 2013), these studies did not control for site‐level differences in isotopic signatures of baseline resources (i.e., plants) which could also vary with disturbance. Ignoring resource isotopic responses to disturbance can lead to erroneous results and interpretations (Hoeinghaus & Zeug, 2008; Post, 2002; Schmidt, Olden, Solomon, & Zanden, 2007). Furthermore, understanding how disturbance affects both the consumer and resource isotopic signatures can offer insight into the mechanisms by which disturbances affect communities and important ecological functions including seed dispersal and predation, aphid tending, top‐down control of insect herbivores, and decomposition and nutrient cycling (Agosti, Majer, Alonso, & Schultz, 2000; Blomqvist, Olff, Blaauw, Bongers, & Putten, 2000; Culver & Beattie, 1980; Dostál, 2005). In our previous work in tallgrass prairies, we document changes in both plant and ant diversity following biomass removal (Kim, Bartel, Wills, Landis, & Gratton, 2018; Kim et al., 2017; Spiesman, Bennett, Isaacs, & Gratton, 2017), in part to due to greater openness and changes in the competitive interactions of ants following the disturbance (Andersen, 2019). These changes in habitat structure and resource availability could also affect the feeding behavior of ants within these grasslands (Kaspari, Donoso, Lucas, Zumbusch, & Kay, 2012). A previous study in disturbed, restored, and remnant pastures in Australia (Gibb & Cunningham, 2011) found that ants fed at lower trophic levels in revegetated pastures, possibility due to greater available of plant sugars, honeydew, and herbivore prey. We predict a similar outcome in trophic structure in harvest sites where habitat openness and subsequent plant productivity are expected to be greater than undisturbed, control sites.

## 2 METHODS

### 2.1 Study system

This study was conducted in tallgrass prairies in southern Wisconsin in 2013–2016. Data from this study were a part of a larger study examining the effects of biomass harvest on predatory arthropod communities and biocontrol services (Kim et al., 2018, 2017). These sites were managed by the United States Fish and Wildlife Service (*N* = 13) and Wisconsin Department of Natural Resources (*N* = 7) and were at least 2 km away from one another. A mixture of perennial grasses (such as *Schizachyrium scoparium*, *Panicum virgatum*, and *Elymus canadensis*) dominated these sites but perennial forbs and legumes such as *Rudbeckia hirta*, *Solidago altissima*, and *Trifolium pratense* were also present (for details on plant communities see Spiesman et al., 2017). While sites varied in size from 12 to 120 hectares, we standardized our ant sampling effort in a 50 m × 50 m area at each site (at least 50 m from any edge to minimize edge effects). Sites were randomly selected to receive at “harvest” treatment at the full site scale whereas the “control” sites were unmanipulated (“harvest” sites, *N* = 9 in 2013; *N* = 10 in 2014 and 2015; “control” sites: *N* = 9 in 2013; *N* = 10 in 2014 and 2015). For the harvest sites, the first biomass harvest occurred in October 2012 at entire site level with standard commercial equipment leaving approximately 30 cm of standing plant residue with all harvestable biomass removed from the site. Biomass was removed annually at the end of the growing season (late September/early October) in 2013–2015. Prior to the experiment, sites were managed via burning and mechanical removal of woody vegetation but the site had not been managed for at least 3 years prior to the start of the experiment.

### 2.2 Insect and plant sampling

Ants were collected once a month in June, July, and August for 3 years (2013–2015) using pitfall traps. At each site, three pitfall traps were established at three permanent sampling stations. Stations were placed at least 50 m from each other to ensure that we were capturing ants across a broad area. Pitfall traps consisted of 1 L deli containers (10 cm diameter opening; Dart Conex®, Mason, MI, USA) filled ¾ full with 50:50 propylene glycol:water solution, placed flush with the ground, and covered with a 6‐mm wire mesh to prevent small mammals and herpetofauna from entering into the traps. Plastic covers (30 cm diameter) were staked 10 cm above the traps to prevent rainwater from flooding the cups. Pitfalls were placed out for 2 weeks continuously during each sampling session. Samples were collected monthly and transferred to 70% ethanol. Upon return to the laboratory, we separated and identified to ants to species, and determined their abundances. Because ethanol can enrich δ^13^C by ~0.61‰ after 6 months (Tillberg et al., 2006), specimens were dried within 6 months after collection. Voucher specimens were pinned and verified with specimens at the Wisconsin Research Insect Collection and the Chicago Field Museum. To determine whether changes in insect herbivore abundances could affect ant feeding, we also sampled insect herbivores at the same time as ant sampling using sweep nets near each of the three sampling stations. At each station, sweep net sampling occurred along 1 m × 50 m belt transects (50 back and forth sweeps per transect) using a 38‐cm diameter sweep net on sunny days with little wind (<5 km/hr). All arthropods classified as herbivores were counted and identified to the family level.

To determine if harvesting could have altered the primary producer (plant) baseline at each site, live plant biomass was collected along a 100 m transect that crossed the middle to the sampling area in 2016. Every 20 m along the transect samples of two plant species, *S. altissima* (tall goldenrod) and *Andropogon gerardi* (big bluestem) were collected by placing out quadrats (30 cm × 30 cm) and harvesting all aboveground biomass of each plant species within the quadrats. These plant species were chosen as indicators of site‐level isotopic basal resource values (plants) because they occurred at all sites in relatively high abundances. We also collected soil samples along the same transects in 2016 to help elucidate mechanisms by which harvest might affect ant trophic structure. Soil samples were collected at 10 cm in depth (after removing top litter layer) using a 1‐inch diameter soil core. Upon returning to the laboratory, plants and soil samples were placed in a drying oven at 60°C for at least 1 week. We sieved soil samples through a 4.75‐mm mesh to remove plant biomass.

### 2.3 Stable isotope sample preparation and analysis

Six ant species (*Aphaenogaster rudis*, *Formica argentea*, *Formica montana*, *Lasius neoniger*, *Myrmica AF‐smi*, and *Myrmica fracticornis*) were selected for stable isotope analysis because they were found in both harvested and control sites in sufficient abundances to achieve the required 1.0 mg sample weight for stable isotope analyses (Banschbach, Brunelle, Bartlett, Grivetti, & Yeamans, 2006; Ellison, Gotelli, Farnsworth, & Alpert, 2012; Lubertazzi, 2012; Maier & Potter, 2005). All six ant species have broad diets and feed as scavengers (*A. rudis*, *F. argentea*, *F. montana*, *L. neoniger*), aphid tenders (*F. montana*, *L. neoniger*, *M. fracticornis*, *M. AF‐smi*), seed predators (*A. rudis*), carnivores (*Myrmica fracticornus*, *M. AF‐smi*), and omnivores (*A. rudis*, *F. argentea*, *L. neoniger*). Ant specimens were dried at 60°C in a drying oven for at least 1 week, ground to a fine powder using a mortar and pestle, then weighed (1 ± 0.2 mg) and packaged in tin capsules (7–9 mm; Costech Analytical Technologies Inc). Each sample contained 3–35 ant specimens depending on their sizes and contained specimens collected from the same trap. If needed, specimens were pooled across sampling stations within each site per sampling session to achieve ~1 mg per tin capsule, resulting in 2–4 replicates (samples) per species per site per year. As a result, for any given site, the isotopic signatures of each ant species were determined from 9 to 12 samples. For each plant species (*S. altissima* and *A. gerardi*), finely ground plant material was packaged into tin capsules (10 mm). Each sample weighed 2.5 mg (±0.05 mg), and there were 3–5 replicates per site per plant species. While different parts of the ant (gaster vs. head/alitrunk) could yield different isotopic signatures representing short‐term (i.e., recently digested) versus long‐term (i.e., tissue integrated) consequences of ant feeding, respectively (Feldhaar, Gebauer, & Blüthgen, 2010), all ant specimens were processed similarly using whole bodies thus allowing us to compare how overall feeding strategies (occurring at both short‐term and long‐term scales) change with harvest.

Packaged samples were sent to the Davis Stable Isotope Facility (University of California) to be analyzed for the stable isotopes, ^13^C and ^15^N, using a PDZ Europa ANCA‐GSL elemental analyzer interfaced to a PDZ Europa 20‐20 isotope mass spectrometer (Sercon Ltd.). Measurements are reported in delta notation (δ) where δ^15^N and δ^13^C = [*R*_sample_/*R*_standard_]) − 1 × 1,000 where *R* is the ratio of the heavy/light isotope content (e.g., ^15^N/^14^N or ^13^C/^12^C). Isotope ratios are expressed in per mil (‰) relative to international reference standards V‐PDB (Vienna PeeDee Belemnite) for carbon and atmospheric nitrogen for nitrogen. Mean *SD*s of the measurement errors on laboratory standards for δ^13^C and δ^15^N were 0.085 and 0.095, respectively. To estimate within‐sample variability, 10% of the *L. neoniger* samples (the most abundant species), 10% of the *S. altissima* and *A. gerardii*, and 10% of soil samples were analyzed in duplicates from which we calculated an average *SD* among replicate samples. Due to limitation in ant biomass, we did not estimate within‐sample variability for all ant species and thus assumed that within‐sample variation was consistent across ant species. Mean *SD* of the duplicate samples of ants was 0.34 for δ^13^C and 0.15 for δ^15^N. Standard deviation of duplicate samples of *S. altissima* was 0.03 for δ^13^C and 0.02 for δ^15^N and *A. gerardi* was 0.08 for δ^13^C and 0.04 for δ^15^N. Standard deviation of duplicate samples of soil was 0.03 for δ^13^C and 0.05 for δ^15^N.

### 2.4 Statistical analyses

Site was the unit of replication, so samples were averaged across sampling sessions and years to yield one value per ant species per site. Preliminary analyses showed that partitioning the data by year and including year as a factor in our model decreased model fit (ΔAIC 18.57); therefore, we averaged data from across all 3 years for each ant species at each site. Because we were often limited in the amount of ant biomass, we did not have enough specimens for all 20 sites so our design was unbalanced (Appendix S1). For plant samples, we were not limited in the amount of plant biomass; therefore, all sites had 3–5 replicates per site for both *S. altissima* and *A. gerardi*.

We quantified the trophic structure of ant communities using three stable isotope‐derived metrics: trophic position, trophic range, and isotopic niche space. Each of these metrics describes different aspects of trophic structure (Layman, Quattrochi, Peyer, Allgeier, & Suding, 2007). Trophic position describes the average number of steps involved in biomass transfer within the food web. Trophic position was as estimated relative to a resource baseline to account for inherent differences among sites in δ^15^N (Post, 2002). Ignoring baseline values and using unadjusted δ^15^N to infer trophic position can lead to erroneous results and interpretation (Post, 2002). We selected *S. altissima* and *A. gerardi* as representative basal resources because they were the most common C3 and C4 plant species, respectively, at our sites and provide a range of food resources for ants. We follow others studies that have used plants as baselines while examining isotopic signatures in arthropods (e.g., Gratton & Denno, 2006; Hoekman, Bartrons, & Gratton, 2012; Ponsard & Arditi, 2000; Roeder & Kaspari, 2017; Woodcock et al., 2013). While we did collect soil at our sites, we did not use soil as our measure of basal resources because small insect and plant fragments, bacteria, and fungi that remained in soil after sieving inflated soil δ^15^N values (at times beyond δ^15^N values of consumer), making the interpretation of ant trophic structure difficult. Therefore, we used the averaged δ^15^N values of *S. altissima* and *A. gerardi* as our basal resource value. The calculation for the trophic position (TP) of a given ant species was TP = *λ* + (δ^15^N_consumer_ − δ^15^N_base_)/Δ*_n_*, where *λ* is the trophic position of the baseline organism (*λ* = 1 for primary producers), δ^15^N_consumer_ is the measured δ^15^N of each ant individual at each site, δ^15^N_base_ is the mean δ^15^N for the baseline plants at each site (Post, 2002). Finally, Δ*_n_* is the enrichment in δ^15^N per trophic level. We assumed an ant‐specific fractionation value of 3.0‰ based on literature (Feldhaar et al., 2010; Post, 2002; Woodcock et al., 2013). Once the TP for each ant sample was calculated, we averaged TP values per ant species across the within‐site replicates.

We also examined how the range in trophic position (hereafter trophic range) might vary with harvest. Trophic range describes the variability of ant feeding responses and is measure that describes the vertical structure of the food web (Layman et al., 2007). Trophic range (TR) of each ant species at a given site was calculated using the coefficient of variation of TP samples collected at a site (*SD* of TP/mean TP, Bluthgen et al., 2003, Young, Jensen, Weidel, & Chandra, 2015 ). This measure of TP is less sensitive to outliers and small sample sizes than conventional measures of trophic ranges (max TP − min TP, Jackson, Inger, Parnell, & Bearhop, 2011). While inter‐ and intra‐annual fluctuations in ant and plant isotopic signatures might be problematic for using plants as basal resources (Iakovlev, Novgorodova, Tiunov, & Reznikova, 2017; Mooney & Tillberg, 2005), we did not detect significant differences in ant signatures across sample years and assume plant signatures were also consistent. Nevertheless, we interpret TP and TR as relative measures of trophic position and trophic range, respectively. Estimating actual TP and TR would require sampling the basal resources concurrently with ants.

To determine how harvest influenced the trophic position and trophic range of ants, we used separate general linear models (GLM) with harvest treatment (control/harvest), ant species, and a harvest treatment × species interaction as fixed effects, and within‐site averaged TP and TR values as the response variables. We also examined how the δ^15^N values of baseline plants and δ^15^N values of ants varied with harvest using GLM with harvest treatment as a fixed effect and within‐site averaged plant δ^15^N and ant δ^15^N as response variables. For plant δ^15^N values, we included soil δ^15^N values as a covariate and a soil × harvest treatment interaction term. For ant δ^15^N values, we also included ant species and harvest treatment × species interaction as fixed effects.

To determine whether isotopic niche space might change with harvest treatment, we used δ^15^N and δ^13^C biplots and performed a permutational analysis of variance, PERMANOVA (adonis function in R). A two‐dimensional isotopic niche space was defined using the δ^15^N and δ^13^C values of each ant species per site standardized by the average baseline values at each site (hereafter Δδ^15^N or Δδ^13^C). Δδ^15^N and Δδ^13^C were calculated as the average isotopic signatures of each ant species per site (δ^15^N or δ^13^C) minus the average isotopic signatures of the two plant species combined (*S. altissima* and *A. gerardi*) at each site. The predictor variables in the PERMANOVA were species and treatment (and interactions) and a Euclidean distance dissimilarity matrix based on the Δδ^15^N and Δδ^13^C was the response variable.

Finally, to help elucidate the mechanisms by which harvest affected ant trophic structure, we performed separate GLMs with harvest as the main fixed effect and ant and insect herbivore abundances as response variables. For ant analyses, we included ant species and a species × harvest treatment term as fixed effects. If significant the species × harvest interaction was significant, we performed post hoc multiple comparison tests to determine how harvest affects each ant species differently. To control for family‐wise error rates typically associated with multiple tests, *p*‐values were adjusted using the Benjamini–Hochberg procedure (Benjamini & Hochberg, 1995). Benjamini–Hochberg critical values were calculated as (*i*/*m*)*Q*, where *i* is the rank, *m* is the total number of tests, and *Q* is the false discovery rate set at 0.05. We also examined relationships between soil δ^15^N, plant δ^15^N, insect herbivore and ant abundances, and trophic structure by performing a series of pair‐wise correlations. All analyses were performed in R 3.5.1 (R Core Team, 2018) with the vegan package (Oksanen et al., 2018).

## 3 RESULTS

### 3.1 Plant and soil isotopic signatures

**Figure 1:**
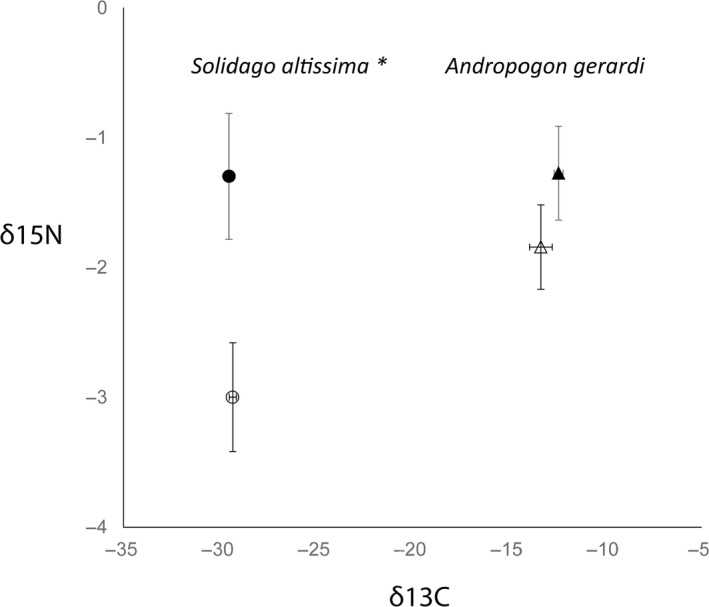
δ^13^C and δ^15^N biplot of *Solidago altissima* (circles) and *Andropogon gerardi* (triangle) in harvest (filled symbols) and control (open symbols) grassland sites. Isotopic values represent averages across all sites. Error bars are ± 1 *SE*. Asterisks denote significant harvest effect

**Figure 2:**
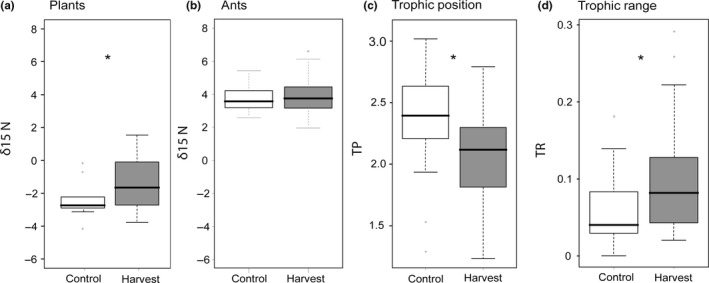
Harvest effects on δ^15^N of (a) baseline plants, (b) ants, (c) tropic position (TP), and (d) trophic range (TR). Isotope values were averaged across species at each site. Boxes represent interquartile ranges, whiskers represent 1.5 times the interquartile range, and solid black lines present median values. Asterisks denote significant harvest effects

**Figure 3:**
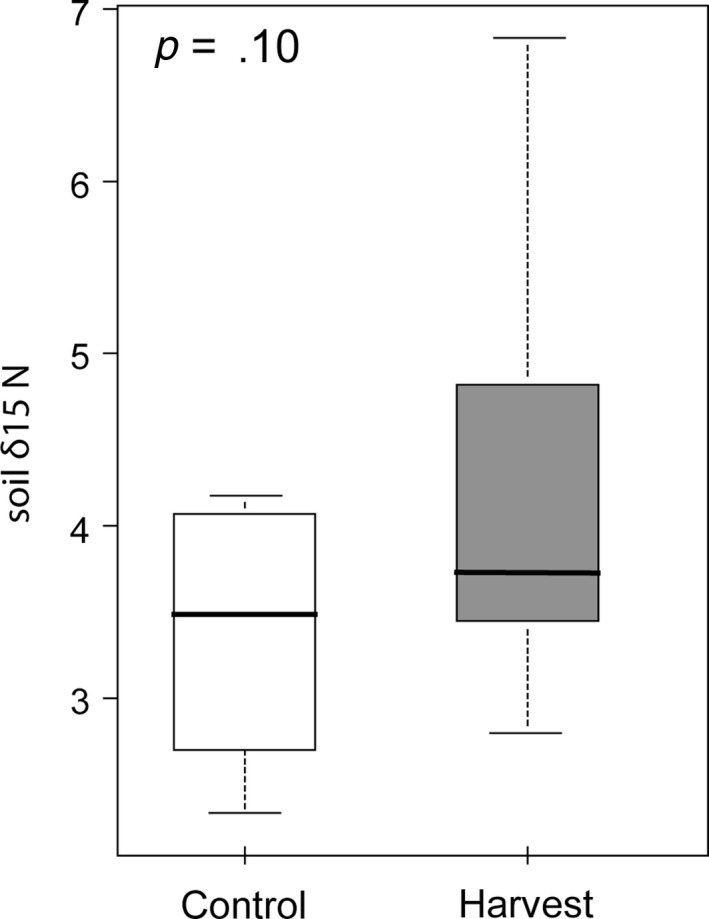
Harvest effects on soil δ^15^N within tallgrass prairies. Soil samples were collected at 10 cm in depth with a 1‐inch diameter soil core. Boxes represent interquartile ranges, whiskers represent 1.5 times the interquartile range, and solid black lines present median values. Values represent average soil δ^15^N values per site

The δ^13^C values of the representative basal resource members (*S. altissima* and *A. gerardi*) varied due to different photosynthetic pathways. *Solidago altissima*, a C3 plant had average δ^13^C values of −29.43‰ whereas *A. gerardi*, a C4 plant, was more enriched with average δ^13^C values of −13.91‰ (Figure 1). In contrast, the δ^15^N isotopic signatures of *S. altissima* and *A. gerardi* were similar averaging −2.41‰ and −1.66‰, respectively. Harvesting enriched plant δ^15^N (*F*_1,14_ = 6.48, *p* = .02, Figure 2a) for both plant species by 56.8% for *S. altissima* and 33.3% for *A. gerardi* but did not affect δ^13^C for either plant species (*F*_1,14_ = 3.00, *p* = .10). Soil δ^15^N did not vary with harvest treatment (*F*_1,16_ = 3.08, *p* = .10, Figure 3a), nor did soil δ^13^C (*F*_1,16_ = 0.76, *p* = .40).

### 3.2 Ant isotopic signatures

**Figure 4:**
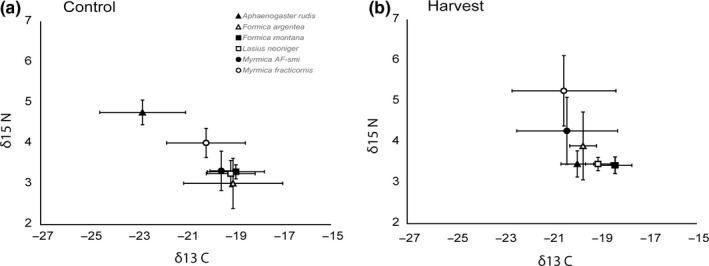
δ^15^N and δ^13^C biplot of grassland ants in control (a) and harvest (b) sites. Values represent isotopic values averaged across all sites. Error bars represent ± 1 *SE*

On average, there were no differences in ant δ^13^C among ant species with average δ^13^C values ranging from −18.9 to −22.11‰ (*F*_5,52_ = 1.2, *p* = .28, Table 1, Figure 4). These δ^13^C values fall within the range of δ^13^C for *S. altissima* and *A. gerardi* suggesting that on average, *S. altissima* and *A. gerardi* were appropriate basal resources to use for TP estimates. In contrast to δ^13^C values, ant δ^15^N varied across ant species (*F*_5,52_ = 4.2, *p* < .01, Appendix S2) with average δ^15^N ranging from 3.3‰ to 4.4‰ within any given site. Moreover, some species showed a wide δ^15^N range within a site (e.g., *F. argentea*: 1.91‰–5.46‰) while others have consistently narrower ranges within a site (e.g., *F. montana*: 2.67‰–4.05‰).

**Table 1:** Isotopic values of δ^13^C and δ^15^N of six ant species in control (A) and harvest (B) sites. Trophic position represents the average number of steps involved in biomass transfer while trophic range describes the variability in trophic position responses. Values represent averages across all sites (±1 *SE*)

Ant species	δ^13^C	δ^15^N	Trophic position	Trophic range
(A) Control				
*Aphaenogaster rudis*	−22.78 (1.77)	4.77 (0.31)	2.81 (0.10)	0.04 (0.01)
*Formica argentea*	−19.03 (2.74)	3.02 (0.62)	2.38 (0.22)	0.07 (0.03)
*Formica montana*	−18.91 (1.32)	3.3 (0.17)	2.15 (0.13)	0.06 (0.01)
*Lasius neoniger*	−19.13 (1.01)	3.26 (0.32)	2.38 (0.08)	0.07 (0.02)
*Myrmica AF‐smi*	−19.52 (0.47)	3.32 (0.48)	2.27 (0.08)	0.04 (0.02)
*Myrmica fracticornis*	−20.15 (2.08)	4.01 (0.36)	2.78 (0.06)	0.06 (0.01)
(B) Harvest				
*Aphaenogaster rudis*	−19.94 (0.68)	3.46 (0.32)	2.63 (0.27)	0.08 (0.02)
*Formica argentea*	−19.7 (0.56)	3.91 (0.83)	2.46 (0.18)	0.05 (0.01)
*Formica montana*	−18.37 (0.69)	3.43 (0.21)	2.05 (0.19)	0.08 (0.02)
*Lasius neoniger*	−19.07 (0.52)	3.46 (0.16)	1.92 (0.20)	0.18 (0.03)
*Myrmica AF‐smi*	−20.37 (2.41)	4.28 (0.95)	2.00 (0.17)	0.06 (<0.01)
*Myrmica fracticornis*	−20.5 (3.57)	5.26 (0.86)	2.23 (0.16)	0.06 (0.02)

The mean trophic position (TP) and range (TR) of ants varied with ant species (TP: *F*_5,52_ = 3.6, *p* < .01, TR: *F*_5,52_ = 2.59, *p* = .03, Table 1, Appendix S2). The numerically dominant *L. neoniger* had a lower trophic position (mean TP = 1.92) than other ant species but had the widest trophic range (TR = 0.18). In contrast, the numerically subordinate *A. rudis* fed at a higher trophic position (TP = 2.81) but had the lowest trophic range (TR = 0.04).

### 3.3 Harvest effects on ant and insect herbivore abundances

**Figure 5:**
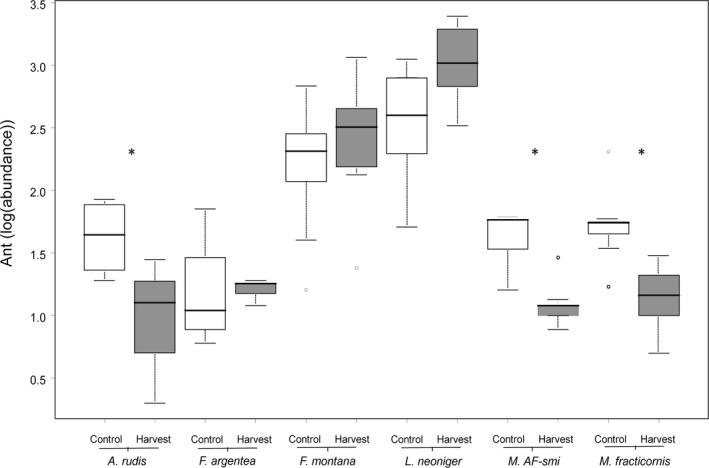
Harvest effects on ant abundances of *Aphaenogaster rudis*, *Formica argentea*, *F. montana*, *Lasius neoniger*, *Myrmica AF‐smi*, and *M. fracticornis*. Ant abundances were averaged across sites and sampled years (2013–2015). Boxes represent interquartile ranges, whiskers represent 1.5 times the interquartile range, and solid black lines present median values. Asterisks represent significant harvest effects after Benjamini–Hochberg *p*‐value corrections

**Figure 6:**
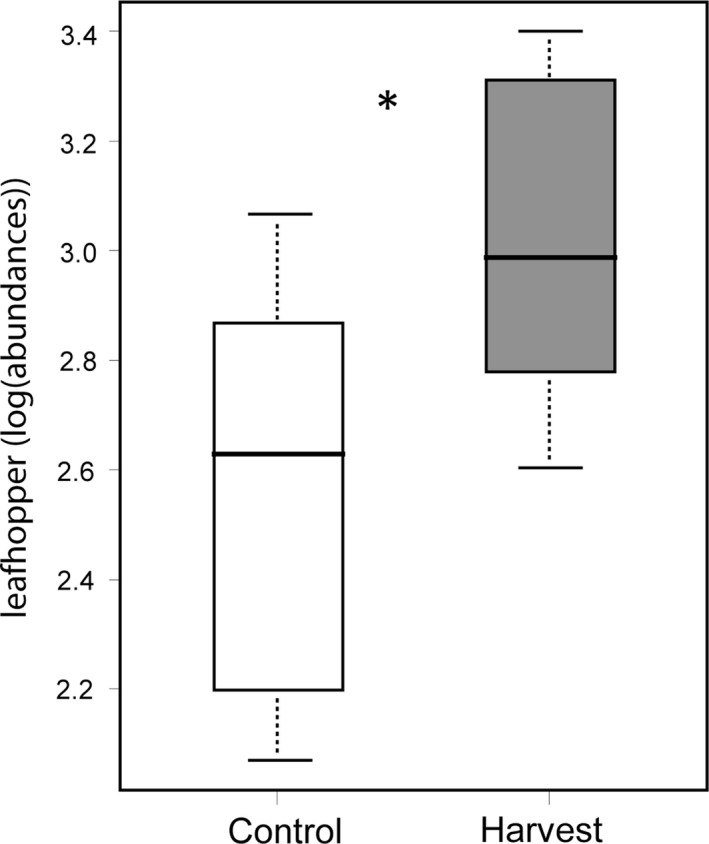
Harvest effects on leafhopper abundances (log‐transformed) in tallgrass prairies. Leafhoppers represented 62% of the captured insect herbivores from 2013 to 2015 using sweep net sampling along 1 m × 50 m transects. Values represent average leafhopper abundances per site. Asterisks denote significant harvest effects

There was a significant interaction between harvest treatment and ant species on ant abundances (*F*_5,52_ = 3.68, *p* < .01, Figure 5). In particular, the two numerically dominant ant species (*L. neoniger* and *F. montana*) were more abundant at harvest sites while the less common species (*A. rudis*, *M. AF‐smi*, and *M. fracticornus*) generally more abundant at control sites. To determine whether differences in ant abundances were in part due to harvest‐mediated changes in insect herbivore abundances, we sampled insect herbivores using sweep net sampling. Leafhopper abundances were the most abundant herbivore making up 62% of the captured individuals at each site. Leafhopper abundances varied with harvest where harvested sites had 60% more leafhoppers than control sites (*F*_1,18_ = 7.22, *p* = .01, Figure 6).

### 3.4 Harvest effects on community‐wide metrics of trophic structure

**Figure 7:**
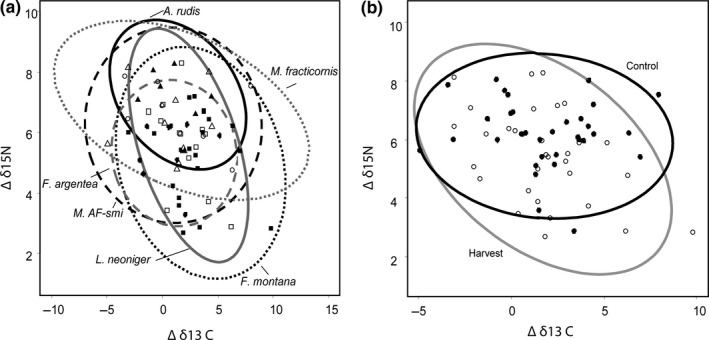
Niche space of ants by species (a) and within harvest and control sites (b). Points represent isotopic values of each ant species averaged across all 3 years by site. Lines represent 95% confidence intervals

Harvest did not affect the δ^15^N signatures of ants (*F*_1,52_ = 0.48, *p* = .48, Figure 2b, Appendix S2). However, once the basal resources were considered, harvest treatment affected trophic position and range (TP: *F*_1,52_ = 5.4, *p* = .02, TR: *F*_1,52_ = 5.84, *p* = .01, Figure 2c,d, Appendix S2). In particular, ants within the harvest treatment fed at lower trophic positions and had wider trophic ranges (average TP 2.15, average TR = 0.10) compared with ants in the control treatment (average TP 2.41, average TR = 0.06). There was no significant interaction between ant species and harvest for trophic position (*F*_5,52_ = 0.54, *p* = .74) or trophic range (*F*_5,52_ = 1.45, *p* = .22) suggesting that the relative trophic structure within the ant communities were maintained with harvest. Finally, we also found that niche space varied with ant species (*F*_1,52_ = 2.81, *p* = .01, Figure 7a) indicating that the different ant species varied with trophic diversity; however, there was no effect of harvest on niche space (*F*_1,52_ = 0.04, *p* = .09, Figure 7b).

### 3.5 Possible mechanisms for trophic structure shifts

**Figure 8:**
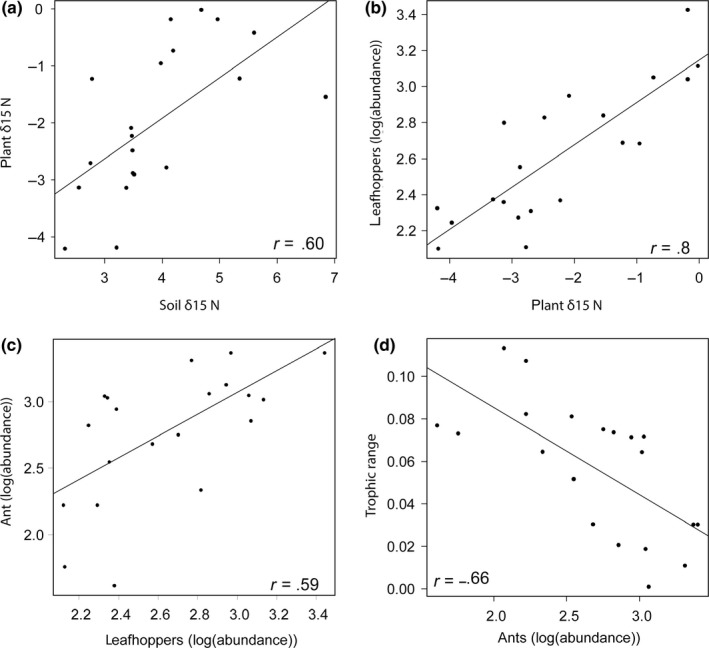
Possible mechanisms for harvest effects on ant trophic structure. (a) Soil δ^15^N relationship with plant δ^15^N, (b) plant δ^15^N relationship with leafhopper abundances (log‐transformed), (c) leafhopper and ant abundances relationship, and (d) relationship of the abundance of numerically dominant ant species (*Lasius neoniger* and *Formica montana*, log‐transformed) and ant trophic range. Each point represents the average value at the site. All correlations were statistically significant *p* < .01

To determine possible mechanisms of harvest effects on the isotopic signatures of ants, we examined relationships between soil δ^15^N, plant δ^15^N, herbivore and ant abundances, and trophic structure. We found positive relationships between soil δ^15^N and plant δ^15^N (*t* = 3.18, *df* = 18, *p* < .01, *r* = .60, Figure 8a) and between plant δ^15^N and leafhopper abundances (*t* = 5.53, *df* = 18, *p* < .01, *r* = .8, Figure 8b) suggesting that soil N might affect plant quality which in turn could attract leafhoppers. We also found a positive relationship between leafhopper and ant abundances (*t* = 3.16, *df* = 18, *p* < .01, Figure 8c) suggesting that sites with more leafhoppers supported more ants. Finally, we found that the abundance of the numerically dominant ant species did not affect ant trophic position (*t* = −1.01, *df* = 18, *p* = 0.33), but their abundances did affect trophic range (*t* = −3.77, *df* = 18, *p* < .01, *r* = −.66, Figure 8d).

## 4 DISCUSSION

We used isotopic signatures to determine how annual harvesting affected the trophic structure and feeding relationships of ants in tallgrass prairies. We found that harvest affected the trophic structure in two different ways: ants fed at lower trophic positions in harvested sites and trophic range was greater in harvested sites suggesting that ants utilized different resources. These changes in TP and TR could be due to harvest‐mediated changes in resource abundance and quality (bottom‐up processes) and/or consumer abundance and community composition (i.e., competition). We discuss each of the possible mechanisms below.

First, harvest effects on trophic structure could be mediated through prey resources. Because these ant species are generalist omnivores, lower trophic positions of ants in harvest sites could suggest that ants are adopting a more “herbivorous” diet composed of more plant‐based food sources such as nectar and seeds or even herbivores rather than other predators. Other studies found similar reduction in TP within disturbed habitats. For example, Gibb and Cunningham (2011) found that ants in revegetated pasture with young trees had lower TP than remnant pastures with older trees and woodlots. Similarly, Reseasco et al. (2012) found that TP varied with habitat fragmentation where that ants within isolated patches had lower TP than ants in connected patches. Both studies attributed lower TPs to the higher availability of plant‐based resources and lower availability of prey in disturbed/isolated sites, resulting in more “herbivorous feeding” strategies of ants feeding plant‐derived resources such as honeydew, plant sugars, and herbivorous prey. In our system, previous work has shown that plant and arthropod communities (Kim et al., 2017; Spiesman et al., 2017) change with harvest where plant, herbivore, and predator abundances increase following repeated biomass removal. Ants could be altering their feeding behavior in response to shifts in resource community structure following harvest. In our study, we found harvest sites had greater leafhopper abundances (the most common herbivore observed in the grasslands) compared with control sites and a positive relationship between leafhopper and ant abundances suggesting that changes in herbivore abundances following harvest could be a mechanism by which harvest impacts ant trophic structure. We also observed increase in TR with harvest suggesting that ant species are broadening their diet breadth to include these herbivore species.

We found species‐level differences in TP and TR but no interaction with harvest, suggesting that the relative TP and TR of each ant species did not change with disturbance. The lack of trophic shift in position and diet breadth among ant species matches previous work with ants and other soil invertebrates following disturbance (Gibb & Cunningham, 2011; Ponsard & Arditi, 2000) suggesting that the trophic roles of ants are conserved. Although our results show relative differences in trophic position and range of ants in the harvest and control sites, they do not tell us specifically what the ants are eating. For example, a more “herbivorous” diet of ants in harvest sites could transpire via feeding on the honeydew produced by leafhoppers or consuming the leafhoppers themselves. Examining the isotopic signatures of other plant species and arthropods in the system could elucidate the exact nature of the feeding relationships (Gratton & Denno, 2006). A mutualistic relationship versus an antagonistic relationship with leafhoppers would have different consequences for the stability of the entire food web community (Sauve, Fontaine, & Thebault, 2013; Thébault & Fontaine, 2010).

Second and related to the mechanism outlined above, harvest effects could be mediated through changes in basal resources. While incorporating isotopic signatures of baseline resources is common in food web studies of aquatic systems, this practice is less common for terrestrial studies. By ignoring the isotopic signatures of baseline resources in food web analyses, we could be underestimating the impact of disturbance on the feeding relationships in ecological communities and overlook possible mechanisms for how TP might change with disturbance. In this study, we observed differences in ant TPs only when we incorporated changes in δ^15^N of baseline resources (plants). Baseline plants were more enriched in δ^15^N at harvest sites and as a consequence, the trophic position of ants (essentially δ^15^N ants–δ^15^N plants) was smaller than control sites. Enrichment of δ^15^N in plants could be due to changes in N cycling and N availability with harvest (Cernusak, Winter, & Turner, 2009). Greater N uptake could be due to greater availability of soil N or greater assimilation rates. Previous studies have found similar results of soil and foliar δ^15^N enrichment following disturbance and have attributed these changes to greater soil organic matter inputs following disturbances such as clear cutting (Knoepp, Taylor, Boring, & Miniat, 2015). However, in our study, we found no difference in soil δ^15^N in control and harvest sites (Figure 3) even though soil and foliar δ^15^N were positively correlated (Figure 6a). This suggests that changes in foliar δ^15^N were not only mediated through soil but though other actions mediated by harvest as well. Greater N assimilation rates in harvest sites might be the mechanism by which plants have greater δ^15^N values (Cernusak et al., 2009; Koch & Fox, 2017). If changes in plant δ^15^N affected plant quality by increasing N availability in leaves (Fang et al., 2011; Hobbie, Macko, & Williams, 2000), then this may explain increases in herbivore abundances following harvest (and subsequent reducing in trophic feeding by ants).

Lastly, harvest effects on trophic structure could be mediated through changes in ant community composition. Ant community composition changed with harvest (Kim et al., 2017, 2018) where harvest sites had greater abundances of the two numerically dominated ant species (*L. neoniger* and *F. montana*) and fewer of the less common ant species (*A. rudis*, *M. AF‐smi*, and *M. fracticornus*). Sites where these two ant species increased in numerical dominance could have increased competitive interactions with other ant species (Anderson, 1992; Andersen & Patel, 1994; Holldobler & Wilson, 1990; Pontin, 1969). As a result, the less common ant species may have truncated diet breadth in response to competition. There was a negative relationship between the average TR of the numerically subordinate species and the abundances of the two numerically dominant ant species across all our sites (Figure 6c) suggesting that diet breadth could be influenced by competition. Similar work has been shown with bee pollinators where in the presence of numerically and behaviorally dominant bees such as honey bees, the diet breadth of native bees was reduced, likely due to competition (Fründ et al., 2013).

## 5 CONCLUSION

We observed changes in the isotopic signatures of ants within tallgrass prairies with harvest suggesting that annual harvesting affects ant trophic structure. In particular, the trophic position of ants was lower in harvest and trophic range increased. Harvest‐mediated changes could be due to changes in plant nutrient assimilation rates, availability of resource prey, or with changes in the ant community composition. Collecting samples from other members of the community would elucidate the exact feeding relationship and help determine the long‐term consequences of feeding shifts on food web stability. Because shifts in resource use can alter energy flow throughout the food web, harvest‐mediated shifts in diet of ants could also affect ecosystem‐level processes such as nutrient cycling. Understanding to what extent shifts in feeding behaviors of ants (and other arthropods) contributes to ecosystem processes is an understudied and promising avenue of research (Yang & Gratton, 2014), integrating concepts from behavioral, community, and ecosystem ecology.

## CONFLICT OF INTEREST

The authors have no conflict of interest to declare.

## AUTHOR CONTRIBUTIONS

TNK and CG conceived and designed the project, TNK and SB executed this study, and TNK, SB, and CG wrote the manuscript (TNK was the main contributor).

## Data Availability

Data are deposited in Dryad (https://doi.org/10.5061/dryad.gc90861).

## References

[ece35523-bib-0001] Agosti, D. , Majer, J. , Alonso, E. , & Schultz, T. R. (2000). Ants: Standard methods for measuring and monitoring biodiversity. Washington, DC: Smithsonian Institution Press.

[ece35523-bib-0002] Andersen, A. N. (1992). Regulation of “momentary” diversity by dominant species in exceptionally rich ant communities of the Australian seasonal tropics. American Naturalist, 140, 401–420. 10.1086/285419 19426050

[ece35523-bib-0003] Andersen, A. N. (2019). Responses of ant communities to disturbance: Five principles for understanding the disturbance dynamics of a globally dominant faunal group. Journal of Animal Ecology, 88, 350–362. 10.1111/1365-2656.12907 30280380

[ece35523-bib-0004] Andersen, A. N. , & Patel, A. D. (1994). Meat ants as dominant members of Australian ant communities: An experimental test of their influence on the foraging success and forager abundance of other species. Oecologia, 98, 15–24. 10.1007/BF00326085 28312791

[ece35523-bib-0005] Banschbach, V. S. , Brunelle, A. , Bartlett, K. M. , Grivetti, J. Y. , & Yeamans, R. L. (2006). Tool use by the forest ant *Aphaenogaster rudis*: Ecology and task allocation. Insectes Sociaux, 53, 463–471. 10.1007/s00040-006-0897-2

[ece35523-bib-0006] Benjamini, Y. , & Hochberg, Y. (1995). Controlling the false discovery rate: A practical and powerful approach to multiple testing. Journal of the Royal Statistical Society B, 57, 289–300.

[ece35523-bib-0007] Biswas, S. R. , & Mallik, A. U. (2010). Disturbance effects on species diversity and functional diversity in riparian and upland plant communities. Ecology, 91, 28–35. 10.1890/08-0887.1 20380192

[ece35523-bib-0008] Blomqvist, M. M. , Olff, H. , Blaauw, M. B. , Bongers, T. , & Putten, W. H. V. D. (2000). Interactions between above‐ and belowground biota: Importance for small‐scale vegetation mosaics in a grassland ecosystem. Oikos, 90, 582–598. 10.1034/j.1600-0706.2000.900316.x

[ece35523-bib-0009] Blüthgen, N. , Gebauer, G. , & Fiedler, K. (2003). Disentangling a rainforest food web using stable isotopes: Dietary diversity in a species‐rich ant community. Oecologia, 137, 426–435. 10.1007/s00442-003-1347-8 12898386

[ece35523-bib-0010] Cardinale, B. J. , & Palmer, M. A. (2002). Disturbance moderates biodiversity–ecosystem function relationships: Experimental evidence from caddisflies in stream mesocosms. Ecology, 83, 1915–1927.

[ece35523-bib-0011] Cernusak, L. A. , Winter, K. , & Turner, B. L. (2009). Plant δ^15^N correlates with the transpiration efficiency of nitrogen acquisition in tropical trees. Plant Physiology, 151, 1667–1676. 19726571 10.1104/pp.109.145870 PMC2773072

[ece35523-bib-0012] Connell, J. H. (1978). Diversity in tropical rain forests and coral reefs. Science, 199, 1302–1310. 17840770 10.1126/science.199.4335.1302

[ece35523-bib-0013] Culver, D. C. , & Beattie, A. J. (1980). The fate of viola seeds dispersed by ants. American Journal of Botany, 67, 710–714. 10.1002/j.1537-2197.1980.tb07701.x

[ece35523-bib-0014] Dayton, P. K. (1971). Competition, disturbance, and community organization: The provision and subsequent utilization of space in a rocky intertidal community. Ecological Monographs, 41, 351–389. 10.2307/1948498

[ece35523-bib-0015] Dostál, P. (2005). Effect of three mound‐building ant species on the formation of soil seed bank in mountain grassland. Flora – Morphology, Distribution, Functional Ecology of Plants, 200, 148–158. 10.1016/j.flora.2004.09.003

[ece35523-bib-0016] Ellison, A. M. , Gotelli, N. J. , Farnsworth, E. J. , & Alpert, G. D. (2012). A field guide to the ants of New England. New Haven, CT: Yale University Press.

[ece35523-bib-0017] Fang, Y. , Yoh, M. , Koba, K. , Zhu, W. , Takebayashi, Y. , Xiao, Y. , … Lu, X. (2011). Nitrogen deposition and forest nitrogen cycling along an urban–rural transect in southern China. Global Change Biology, 17, 872–885. 10.1111/j.1365-2486.2010.02283.x

[ece35523-bib-0018] Feldhaar, H. , Gebauer, G. , & Blüthgen, N. (2010). Stable isotopes: Past and future in exposing secrets of ant nutrition (Hymenoptera: Formicidae). Myrmecological News, 13, 3–13.

[ece35523-bib-0019] Fiedler, K. , Kuhlmann, F. , Schlick‐Steiner, B. C. , Steiner, F. M. , & Gebauer, G. (2007). Stable N‐isotope signatures of central European ants – assessing positions in a trophic gradient. Insectes Sociaux, 54, 393–402. 10.1007/s00040-007-0959-0

[ece35523-bib-0020] Fründ, J. , Dormann, C. F. , Holzschuh, A. , & Tscharntke, T. (2013). Bee diversity effects on pollination depend on functional complementarity and niche shifts. Ecology, 94, 2042–2054. 10.1890/12-1620.1 24279275

[ece35523-bib-0021] Fry, B. (2006). Stable isotope ecology. New York, NY: Springer.

[ece35523-bib-0022] Gibb, H. , & Cunningham, S. A. (2011). Habitat contrasts reveal a shift in the trophic position of ant assemblages. Journal of Animal Ecology, 80, 119–127. 10.1111/j.1365-2656.2010.01747.x 20831728

[ece35523-bib-0023] Gratton, C. , & Denno, R. F. (2006). Arthropod food web restoration following removal of an invasive wetland plant. Ecological Applications, 16, 622–631. 10.1890/1051-0761(2006)016[0622:AFWRFR]2.0.CO;2 16711049

[ece35523-bib-0024] Hobbie, E. A. , Macko, S. A. , & Williams, M. (2000). Correlations between foliar δ^15^N and nitrogen concentrations may indicate plant‐mycorrhizal interactions. Oecologia, 122, 273–283. 10.1007/PL00008856 28308382

[ece35523-bib-0025] Hoeinghaus, D. J. , & Zeug, S. C. (2008). Can stable isotope ratios provide for community‐wide measures of trophic structure? Comment. Ecology, 89, 2353–2357. 10.1890/07-1143.1 18724745

[ece35523-bib-0026] Hoekman, D. , Bartrons, M. , & Gratton, C. (2012). Ecosystem linkages revealed by experimental lake‐derived isotope signal in heathland food webs. Oecologia, 170(3), 735–743. 10.1007/s00442-012-2329-5 22526944

[ece35523-bib-0027] Hölldobler, B. , & Wilson, E. O. (1990). The ants. London, UK: Springer‐Verlag.

[ece35523-bib-0028] Iakovlev, I. K. , Novgorodova, T. A. , Tiunov, A. V. , & Reznikova, Z. I. (2017). Trophic position and seasonal changes in the diet of the red wood ant *Formica aquilonia* as indicated by stable isotope analysis. Ecological Entomology, 42, 263–272.

[ece35523-bib-0029] Inouye, D. W. (1978). Resource partitioning in Bumblebees: Experimental studies of foraging behavior. Ecology, 59, 672–678. 10.2307/1938769

[ece35523-bib-0030] Jackson, A. L. , Inger, R. , Parnell, A. C. , & Bearhop, S. (2011). Comparing isotopic niche widths among and within communities: SIBER – Stable isotope Bayesian Ellipses in R. Journal of Animal Ecology, 80, 595–602. 10.1111/j.1365-2656.2011.01806.x 21401589

[ece35523-bib-0031] Jaworski, C. C. , Bompard, A. , Genies, L. , Amiens‐Desneux, E. , & Desneux, N. (2013). Preference and prey switching in a generalist predator attacking local and invasive alien pests. PLoS ONE, 8, e82231 10.1371/journal.pone.0082231 24312646 PMC3846826

[ece35523-bib-0032] Kaspari, M. , Donoso, D. , Lucas, J. A. , Zumbusch, T. , & Kay, A. D. (2012). Using nutritional ecology to predict community structure: A field test in neotropical ants. Ecosphere, 3, 1–15. 10.1890/ES12-00136.1

[ece35523-bib-0033] Kim, T. N. , Bartel, S. , Wills, B. D. , Landis, D. A. , & Gratton, C. (2018). Disturbance differentially affects alpha and beta diversity of ants in tallgrass prairies. Ecosphere, 9, e02399 10.1002/ecs2.2399

[ece35523-bib-0034] Kim, T. N. , Fox, A. F. , Wills, B. D. , Meehan, T. D. , Landis, D. A. , & Gratton, C. (2017). Harvesting biofuel grasslands has mixed effects on natural enemy communities and no effects on biocontrol services. Journal of Applied Ecology, 54(6), 2011–2021. 10.1111/1365-2664.12901

[ece35523-bib-0035] Knoepp, J. D. , Taylor, S. R. , Boring, L. R. , & Miniat, C. F. (2015). Influence of forest disturbance on stable nitrogen isotope ratios in soil and vegetation profiles. Soil Science Society of America Journal, 79(5), 1470 10.2136/sssaj2015.03.0101

[ece35523-bib-0036] Koch, P. L. , & Fox, L. R. (2017). Browsing impacts on the stable isotope composition of chaparral plants. Ecosphere, 8, e01686 10.1002/ecs2.1686

[ece35523-bib-0037] Layman, C. A. , Quattrochi, J. P. , Peyer, C. M. , Allgeier, J. E. , & Suding, K. (2007). Niche width collapse in a resilient top predator following ecosystem fragmentation. Ecology Letters, 10, 937–944. 17845294 10.1111/j.1461-0248.2007.01087.x PMC2040226

[ece35523-bib-0038] Lubertazzi, D. (2012). The biology and natural history of *Aphaenogaster rudis* . Psyche: A Journal of Entomology, 2012, 1–11.

[ece35523-bib-0039] Maier, R. M. , & Potter, D. A. (2005). Factors affecting distribution of the mound‐building ant *Lasius neoniger* (Hymenoptera: Formicidae) and implications for management on golf course putting greens. Journal of Economic Entomology, 98, 891–898. 16022318 10.1603/0022-0493-98.3.891

[ece35523-bib-0040] Markl, J. S. , Schleuning, M. , Forget, P. M. , Jordano, P. , Lambert, J. E. , Traveset, A. , … Böhning‐Gaese, K. (2012). Meta‐analysis of the effects of human disturbance on seed dispersal by animals. Conservation Biology: The Journal of the Society for Conservation Biology, 26, 1072–1081. 10.1111/j.1523-1739.2012.01927.x 22971077

[ece35523-bib-0041] McCann, K. S. (2000). The diversity – stability debate. Nature, 405, 228–233. 10.1038/35012234 10821283

[ece35523-bib-0042] Mooney, K. A. , & Tillberg, C. V. (2005). Temporal and spatial variation to ant omnivory in pine forests. Ecology, 86, 1225–1235. 10.1890/04-0938

[ece35523-bib-0043] Murdoch, W. W. (1969). Switching in general predators: Experiments on predator specificity and stability of prey populations. Ecological Monographs, 39, 335–354. 10.2307/1942352

[ece35523-bib-0044] Oksanen, J. , Blanchet, F. G. , Kindt, R. , Legendre, P. , Minchin, P. R. , O'Hara, R. B. , … Wagner, H. (2018). vegan: Community ecology package. R package version 3.3.1.

[ece35523-bib-0045] Ottonetti, L. , Tucci, L. , Chelazzi, G. , & Santini, G. (2008). Stable isotopes analysis to assess the trophic role of ants in a Mediterranean agroecosystem. Agricultural and Forest Entomology, 10, 29–36. 10.1111/j.1461-9563.2007.00358.x

[ece35523-bib-0046] Pacala, S. , & Roughgarden, J. (1982). Resource partitioning and interspecific competition in two two‐species insular Anolis lizard communities. Science, 217(4558), 444–446. 17782979 10.1126/science.217.4558.444

[ece35523-bib-0047] Penick, C. A. , Savage, A. M. , & Dunn, R. R. (2015). Stable isotopes reveal links between human food inputs and urban ant diets. Proceedings of the Royal Society B: Biological Sciences, 282, 20142608 10.1098/rspb.2014.2608 PMC4426608 25833850

[ece35523-bib-0048] Ponsard, S. , & Arditi, R. (2000). What can stable isotopes (δ^15^N and δ^13^C) tell about the food web of soil macro‐invertebrates? Ecology, 81, 852–864.

[ece35523-bib-0049] Pontin, A. J. (1969). Experimental transplantation of nest‐mounds of the ant *Lasius flavus* (F.) in a habitat containing also *L. niger* (L.) and *Myrmica scabrinodis* Nyl. Journal of Animal Ecology, 38, 747–754. 10.2307/3044

[ece35523-bib-0050] Post, D. M. (2002). Using stable isotopes to estimate trophic position: Models, methods, and assumptions. Ecology, 83, 703–718. 10.1890/0012-9658(2002)083[0703:USITET]2.0.CO;2

[ece35523-bib-0051] R Core Team (2018). R: A language and environment for statistical computing. Vienna, Austria: R Foundation for Statistical Computing Retrieved from https://www.R-project.org/

[ece35523-bib-0052] Resasco, J. , Levey, D. J. , & Damschen, E. I. (2012). Habitat corridors alter relative trophic position of fire ants. Ecosphere, 3, art100 10.1890/ES12-00266.1

[ece35523-bib-0053] Roeder, K. A. , & Kaspari, M. (2017). From cryptic herbivore to predator: Stable isotopes reveal consistent variability in trophic levels in an ant population. Ecology, 98, 297–303. 10.1002/ecy.1641 28052342

[ece35523-bib-0054] Sauve, A. M. , Fontaine, C. , & Thebault, E. (2013). Structure‐stability relationships in networks combining mutualistic and antagonistic interactions. Oikos, 123, 378–384. 10.1111/j.1600-0706.2013.00743.x

[ece35523-bib-0055] Schmidt, S. N. , Olden, J. D. , Solomon, C. T. , & Zanden, M. J. V. (2007). Quantitative approaches to the analysis of stable isotope food web data. Ecology, 88, 2793–2802. 10.1890/07-0121.1 18051648

[ece35523-bib-0056] Sousa, W. P. (1984). The role of disturbance in natural communities. Annual Review of Ecology and Systematics, 15, 353–391. 10.1146/annurev.es.15.110184.002033

[ece35523-bib-0057] Spiesman, B. J. , Bennett, A. , Isaacs, R. , & Gratton, C. (2017). Bumble bee colony growth and reproduction depend on local flower dominance and natural habitat area in the surrounding landscape. Biological Conservation, 206, 217–223. 10.1016/j.biocon.2016.12.008

[ece35523-bib-0058] Spiesman, B. J. , & Gratton, C. (2016). Flexible foraging shapes the topology of plant–pollinator interaction networks. Ecology, 97, 1431–1441. 10.1890/15-1735.1 27459774

[ece35523-bib-0059] Thébault, E. , & Fontaine, C. (2010). Stability of ecological communities and the architecture of mutualistic and trophic networks. Science, 329, 853–856. 10.1126/science.1188321 20705861

[ece35523-bib-0060] Thom, D. , & Seidl, R. (2016). Natural disturbance impacts on ecosystem services and biodiversity in temperate and boreal forests. Biological Reviews, 91, 760–781. 10.1111/brv.12193 26010526 PMC4898621

[ece35523-bib-0061] Tillberg, C. V. , McCarthy, D. P. , Dolezal, A. G. , & Suarez, A. V. (2006). Measuring the trophic ecology of ants using stable isotopes. Insectes Sociaux, 53, 65–69. 10.1007/s00040-005-0836-7

[ece35523-bib-0062] Tilman, D. , Reich, P. , Phillips, H. , Menton, M. , Patel, A. , Vos, E. , … Knops, J. (2000). Fire suppression and ecosystem carbon storage. Ecology, 81, 2680–2685. 10.1890/0012-9658(2000)081[2680:FSAECS]2.0.CO;2

[ece35523-bib-0063] Vander Zanden, M. J. , Casselman, J. M. , & Rasmussen, J. B. (1999). Stable isotope evidence for the food web consequences of species invasions in lakes. Nature, 401, 464–467. 10.1038/46762

[ece35523-bib-0064] Vander Zanden, M. J. , Olden, J. D. , Gratton, C. , & Tunney, T. D. (2016). Food web theory and ecological restoration In Palmer M. A., Zedler J. B., & Falk D. A. (Eds.), Foundations of restoration ecology (2nd ed.). Washington, DC: Island Press/Center for Resource Economics.

[ece35523-bib-0065] Woodcock, P. , Edwards, D. P. , Newton, R. J. , Khen, C. V. , Bottrell, S. H. , & Hamer, K. C. (2013). Impacts of intensive logging on the trophic organisation of ant communities in a biodiversity hotspot. PLoS ONE, 8, e60756 10.1371/journal.pone.0060756 23593302 PMC3622666

[ece35523-bib-0066] Wootton, J. T. (1998). Effects of disturbance on species diversity: A multitrophic perspective. American Naturalist, 152, 803–825. 10.1086/286210 18811429

[ece35523-bib-0067] Yang, L. H. , & Gratton, C. (2014). Insects as drivers of ecosystem processes. Current Opinion in Insect Science, 2, 26–32. 10.1016/j.cois.2014.06.004 32846721

[ece35523-bib-0068] Young, T. , Jensen, O. P. , Weidel, B. C. , & Chandra, S. (2015). Natural trophic variability in a large, oligotrophic, near-pristine lake. Journal of Great Lakes Research, 41, 463–472.

