# Supplementary material for: Grassland harvesting alters ant community trophic structure: An isotopic study in tallgrass prairies
Source: Ecol Evol. 2019 Aug 13;9(17):9815–26. doi: 10.1002/ece3.5523 (PMC6745673; doi:10.1002/ece3.5523)
Supplement: Supplementary file 1 [file ECE3-9-9815-s001.docx]

**Supplemental Information**

Grassland harvesting alters ant community trophic structure: an isotopic study in tallgrass prairies

Tania Kim^1*^, Savannah Bartel^2^, and Claudio Gratton^1,3^

^1^University of Wisconsin Madison, Great Lakes Bioenergy Research Center, Madison Wisconsin 53726, USA. ^2^University of Wisconsin Madison, Department of Integrative Biology, Madison Wisconsin 53706, USA. ^3^University of Wisconsin Madison, Department of Entomology, Madison Wisconsin 53706, USA

***Corresponding author**: tkim@ksu.edu; 785-532-4709 (office)

Current address: Kansas State University, Department of Entomology, Manhattan, KS 66506 USA.

**Email addresses co-authors**: Savannah Bartel (bartel2@wisc.edu); Claudio Gratton (cgratton@wisc.edu)

**Supporting Information**

**Appendix S1.** Number of tallgrass prairie sites in southern Wisconsin, USA where ant specimens were collected for this study.

|  |  | |
| --- | --- | --- |
| Species | Control | Harvest |
| *Formica montana* | 9 | 8 |
| *Lasius neoniger* | 5 | 7 |
| *Aphaenogaster rudis* | 4 | 3 |
| *Formica argentea* | 4 | 5 |
| *Myrmica AF-smi* | 5 | 5 |
| *Myrmica fracticornis* | 4 | 3 |

**Appendix S2.** Harvest and species level effects on (a) δ^15^N of plants, (b) δ^15^N of ants, (c) trophic position, and (d) trophic range. All response variables were averaged at the site level.

| a) δ^15^N Plants | Df | SS | MS | F | P |
| --- | --- | --- | --- | --- | --- |
| Treatment | 1 | 6.33 | 6.33 | 6.48 | 0.02 |
| Soil δ^15^N | 1 | 5.28 | 5.28 | 5.40 | 0.03 |
| Soil x Treatment | 1 | 3.73 | 3.72 | 3.82 | 0.07 |
| Residuals | 14 | 13.66 | 0.97 |  |  |
|  |  |  |  |  |  |
| b) δ^15^N ants | Df | SS | MS | F | P |
| Treatment | 1 | 0.39 | 0.39 | 0.48 | 0.48 |
| Species | 5 | 17.42 | 3.4 | 4.2 | < 0.01 |
| Treatment x Species | 5 | 1.26 | 0.25 | 0.31 | 0.90 |
| Residuals | 52 | 42.39 | 0.82 |  |  |
|  |  |  |  |  |  |
| c) Trophic position | Df | SS | MS | F | P |
| Treatment | 1 | 0.99 | 0.98 | 5.4 | 0.02 |
| Species | 5 | 3.31 | 0.66 | 3.6 | < 0.01 |
| Treatment x Species | 5 | 0.49 | 0.09 | 0.54 | 0.74 |
| Residuals | 52 | 9.45 | 0.18 |  |  |
|  |  |  |  |  |  |
| d) Trophic range | Df | SS | MS | F | P |
| Treatment | 1 | 0.01 | 0.02 | 5.84 | 0.01 |
| Species | 5 | 0.04 | <0.01 | 2.59 | 0.03 |
| Treatment x Species | 5 | 0.02 | < 0.01 | 1.45 | 0.22 |
| Residuals | 52 | 0.17 | < 0.01 |  |  |
|  |  |  |  |  |  |
